# Supplementary figures and images for: Pine pollen reverses the function of hepatocellular carcinoma by inhibiting α-Enolase mediated PI3K/AKT signaling pathway
Source: PLoS One. 2024 Nov 22;19(11):e0312434. doi: 10.1371/journal.pone.0312434 (PMC11584142; doi:10.1371/journal.pone.0312434)

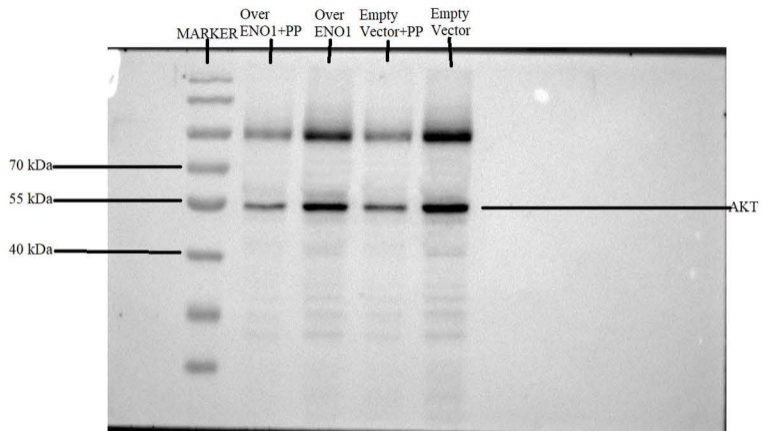

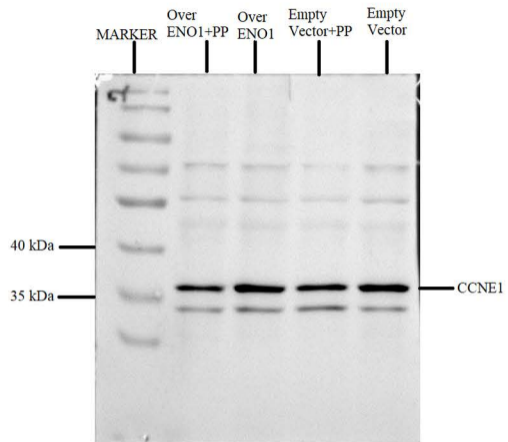

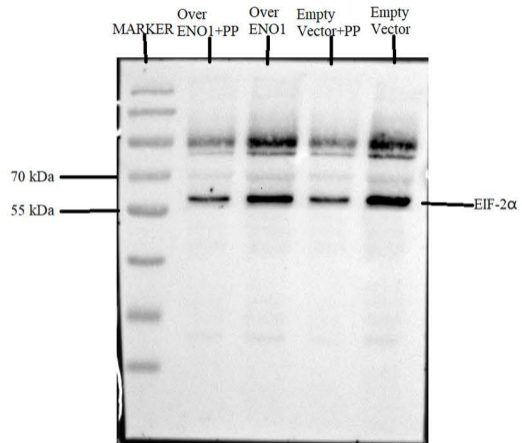

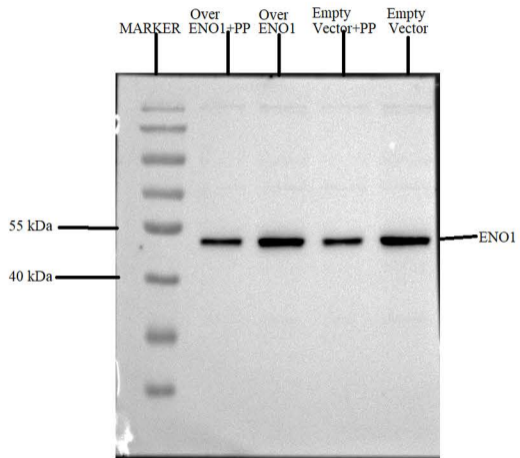

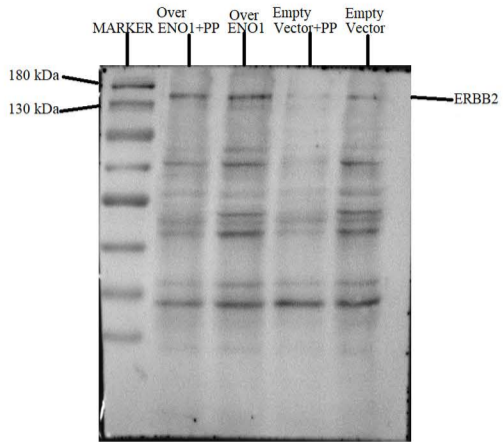

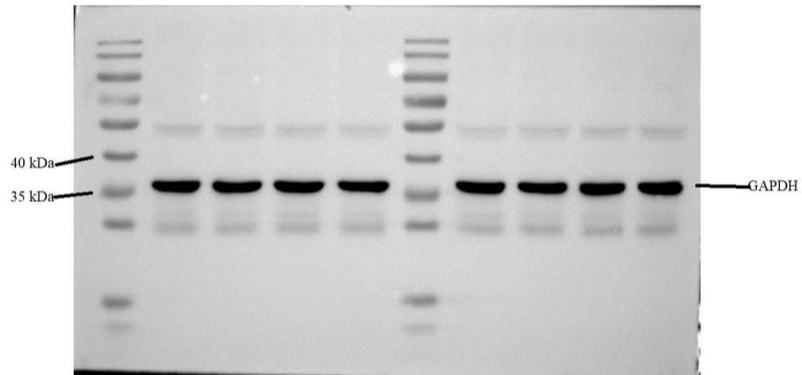

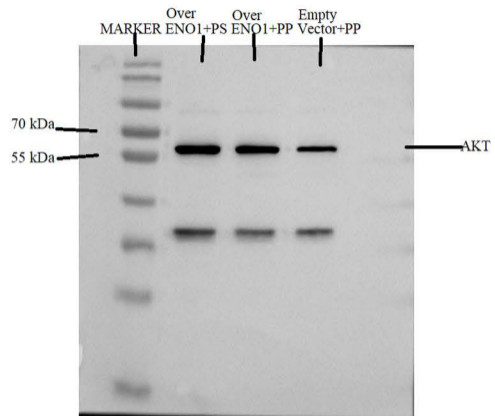

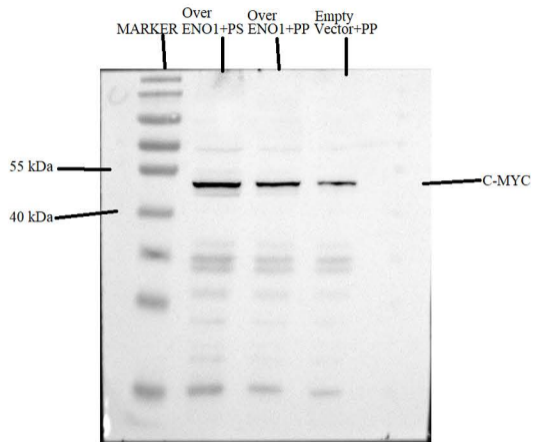

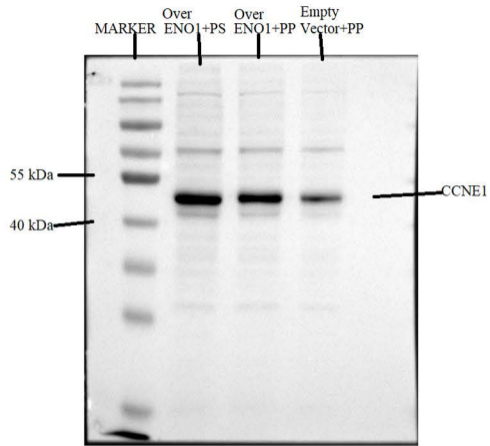

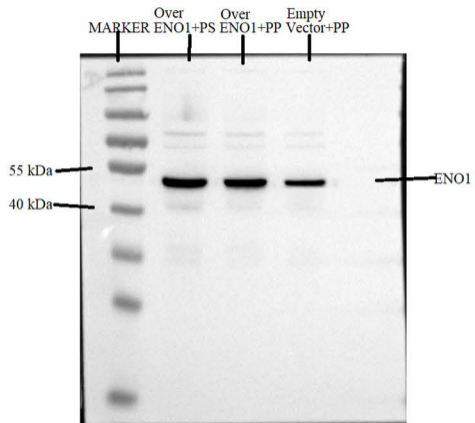

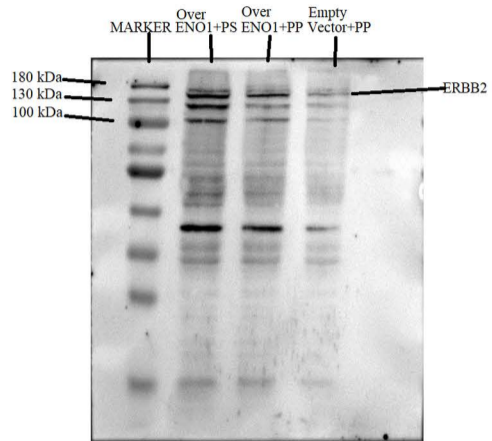

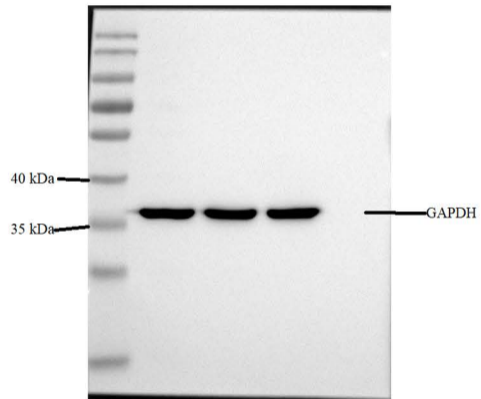

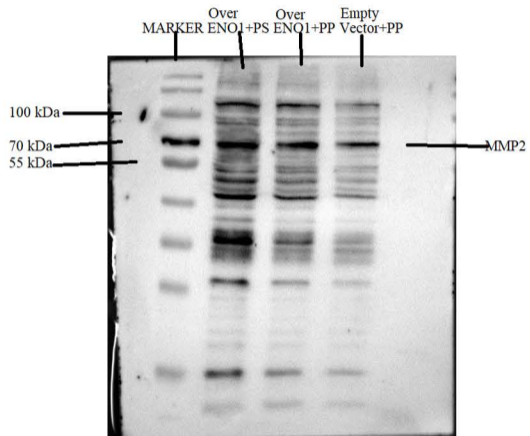

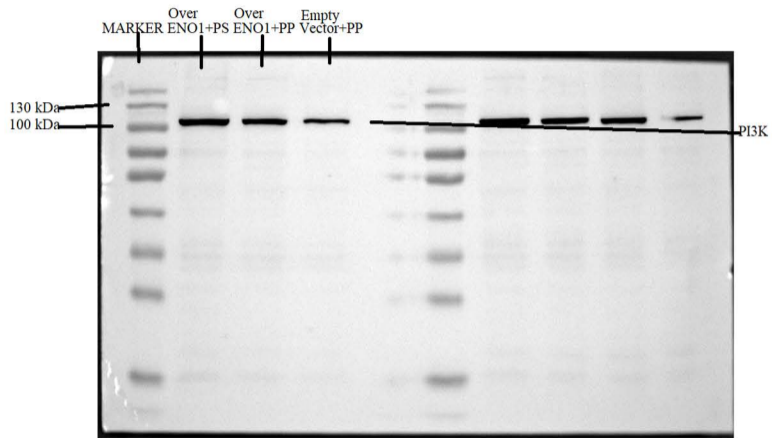

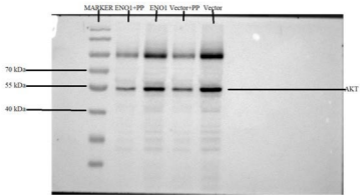

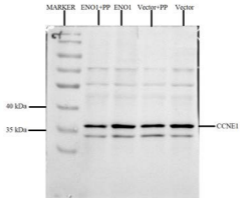

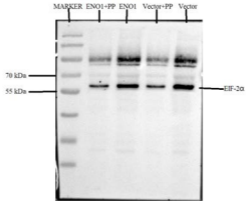

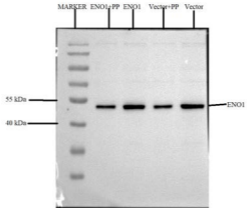

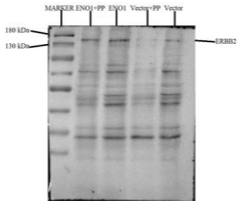

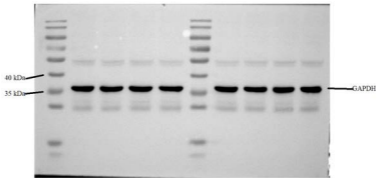

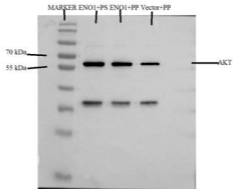

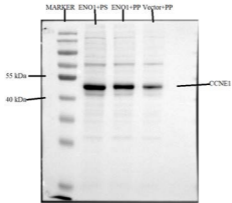

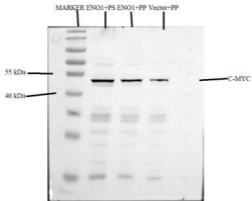

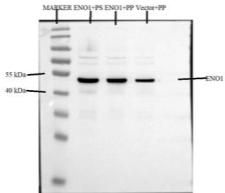

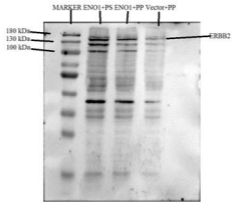

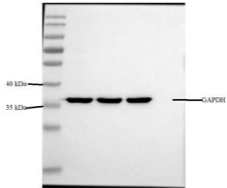

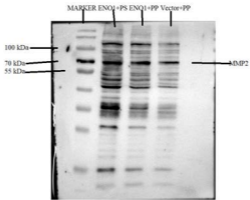

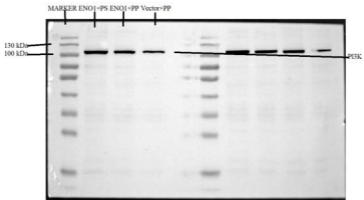

Supplement: S1 File — The raw images of Western blot. (PDF) [file pone.0312434.s001.pdf]

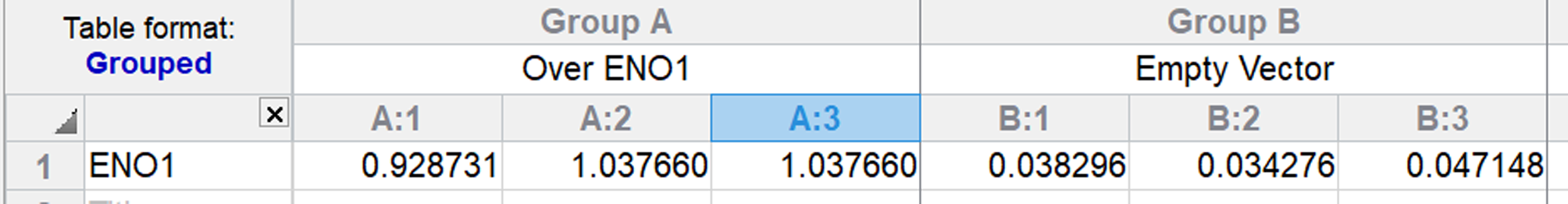

Supplement: S3 File — The software of test data. (ZIP) [file pone.0312434.s003.zip › Figure 4 (E)'.tif]

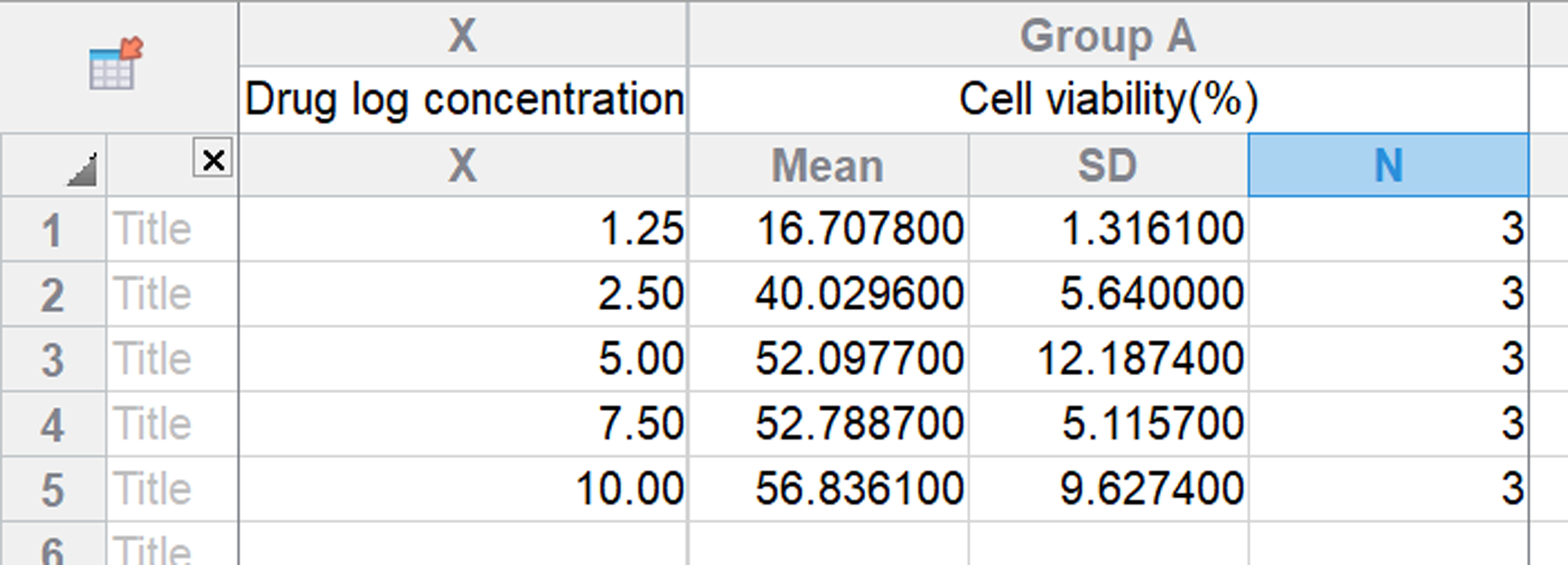

Supplement: S3 File — The software of test data. (ZIP) [file pone.0312434.s003.zip › Figure 5 (C)'.tif]

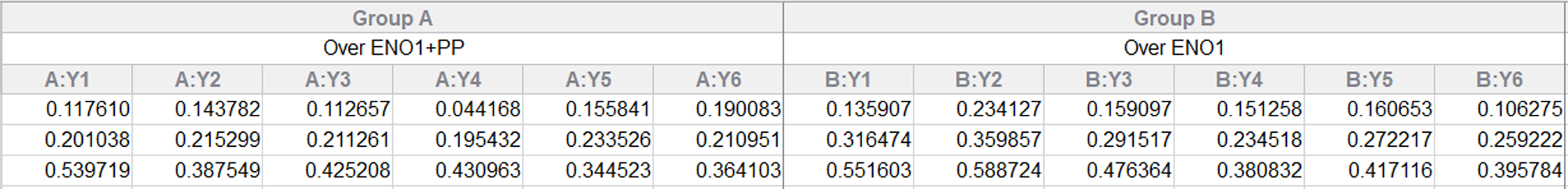

Supplement: S3 File — The software of test data. (ZIP) [file pone.0312434.s003.zip › Figure 7(F)-1'.tif]

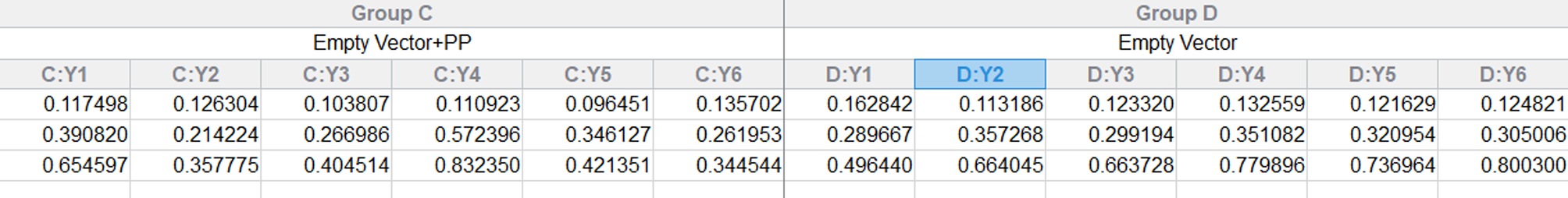

Supplement: S3 File — The software of test data. (ZIP) [file pone.0312434.s003.zip › Figure 7(F)-2'.tif]

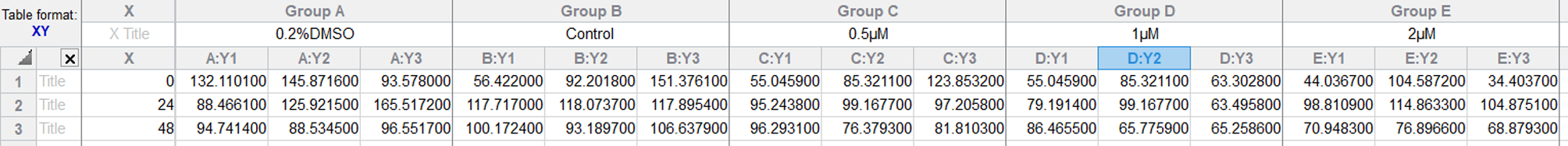

Supplement: S3 File — The software of test data. (ZIP) [file pone.0312434.s003.zip › Figure 8 (A)'.tif]

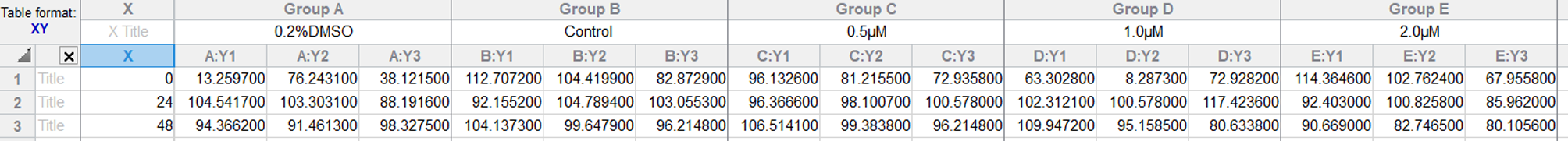

Supplement: S3 File — The software of test data. (ZIP) [file pone.0312434.s003.zip › Figure 8(B)'.tif]

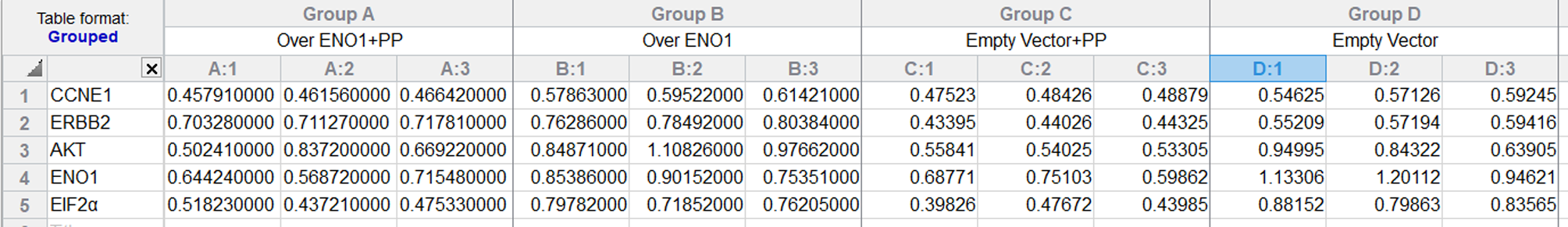

Supplement: S3 File — The software of test data. (ZIP) [file pone.0312434.s003.zip › Figure 9 (B)'.tif]

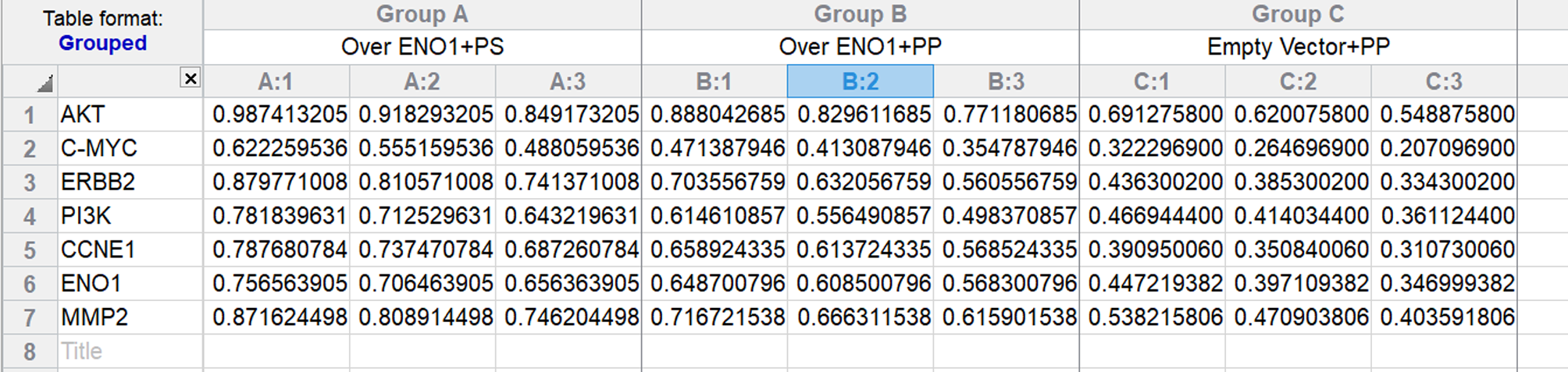

Supplement: S3 File — The software of test data. (ZIP) [file pone.0312434.s003.zip › Figure 9 (D)'.tif]

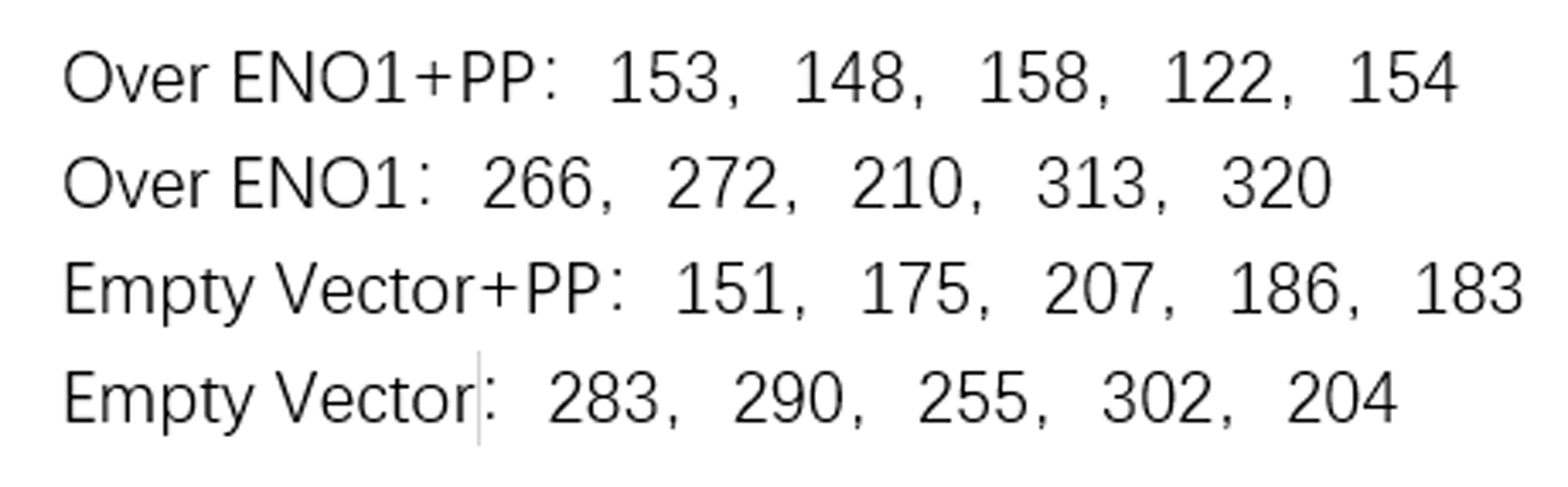

Supplement: S3 File — The software of test data. (ZIP) [file pone.0312434.s003.zip › Table 1'.tif]
