# Supplementary material for: Pine pollen reverses the function of hepatocellular carcinoma by inhibiting α-Enolase mediated PI3K/AKT signaling pathway
Source: PLoS One. 2024 Nov 22;19(11):e0312434. doi: 10.1371/journal.pone.0312434 (PMC11584142; doi:10.1371/journal.pone.0312434)
Supplement: S5 File — The Original data in the table. (DOCX) [file pone.0312434.s005.docx]

Table 1 data

Over ENO1+PP: 153, 148, 158, 122, 154

Over ENO1: 266, 272, 210, 313, 320

Empty Vector+PP: 151, 175, 207, 186, 183

Empty Vector: 283, 290, 255, 302, 204

Table 2 data

Over ENO1+PP:

12h 0.117610, 0.143782, 0.112657, 0.044168, 0.155841, 0.190083

24h 0.201038, 0.215299, 0.211261, 0.195432, 0.233526, 0.210951

48h 0.539719, 0.387549, 0.425208, 0.430963, 0.344523, 0.364103

Over ENO1:

12h 0.135907, 0.234127, 0.159097, 0.151258, 0.160653, 0.106275

24h 0.316474, 0.359857, 0.291517, 0.234518, 0.272217, 0.259222

48h 0.551603, 0.588724, 0.476364, 0.380832, 0.417116, 0.395784

Empty Vector+PP:

12h 0.117498, 0.126304, 0.103807, 0.110923, 0.096451, 0.135702

24h 0.390820, 0.214224, 0.266986, 0.572396, 0.346127, 0.261953

48h 0.654597, 0.357775, 0.404514, 0.832350, 0.421351, 0.344544

Empty Vector:

12h 0.162842, 0.113186, 0.123320, 0.132559, 0.121629, 0.124821

24h 0.289667, 0.357268, 0.299194, 0.351082, 0.320954, 0.305006

48h 0.496440, 0.664045, 0.663728, 0.779896, 0.736964, 0.800300

Table 3 data

ALT

Over ENO1+PP: 350.5 394.5 337.4 407.6 347.3 397.7

over ENO2+Ps: 493.6 566 499.3 560.3 502.7 556.9

empty +PP: 79.2 113.6 83.6 109.2 78.3 114.5

blank: 106.3 75.9 75.1 107.1 104 78.2

AST

over ENO1+PP: 555.6 620.4 539.8 636.2 541.9 634.1

over ENO2+Ps: 955 1067 945.8 1076.2 968 1054

empty +PP: 202.3 246.5 206.5 242.3 208.6 240.2

blank: 290.3 251.9 247 295.2 288.3 253.9

AST/ALT

over ENO1+PP:

| 1.5851641 | 1.572624 | 1.599881 | 1.560844 | 1.560322 | 1.594417903 |
| --- | --- | --- | --- | --- | --- |

over ENO2+Ps:

| 1.934765 | 1.885159 | 1.894252 | 1.920757 | 1.925602 | 1.89261986 |
| --- | --- | --- | --- | --- | --- |

empty +PP:

| 2.5542929 | 2.169894 | 2.470096 | 2.218864 | 2.664112 | 2.097816594 |
| --- | --- | --- | --- | --- | --- |

Blank:

| 2.7309501 | 3.318841 | 3.288948 | 2.756303 | 2.772115 | 3.246803069 |
| --- | --- | --- | --- | --- | --- |

Tumor weight

over ENO1+PP: 0.0802 0.0926 0.0779 0.0949 0.0773 0.0955

over ENO1+Ps: 0.2614 0.241 0.2312 0.2712 0.2252 0.2772

empty +PP: 0.0515 0.0637 0.0501 0.0651 0.0528 0.0624

Table 4 data

Cyclin E1

Over ENO1 + PP: 0.45791, 0.46156, 0.46642

Over ENO1: 0.57863, 0.59522, 0.61421

Empty Vector + PP: 0.47523, 0.48426, 0.48879

Empty Vector: 0.54625, 0.57126, 0.59245

ERBB2

Over ENO1 + PP: 0.70328, 0.71127, 0.71781

Over ENO1: 0.76286, 0.78492, 0.80384

Empty Vector + PP: 0.43395, 0.44026, 0.44325

Empty Vector: 0.55209, 0.57194, 0.59416

Table 5 data

AKT

Over ENO1 + PP: 0.50241, 0.8372, 0.66922

Over ENO1: 0.84871, 1.10826, 0.97662

Empty Vector + PP: 0.55841, 0.54025, 0.53305

Empty Vector: 0.94995, 0.84322, 0.63905

ENO1

Over ENO1 + PP: 0.64424, 0.56872, 0.71548

Over ENO1: 0.85386, 0.90152, 0.75351

Empty Vector + PP: 0.68771, 0.75103, 0.59862

Empty Vector: 1.13306, 1.20112, 0.94621

EIF2 α

Over ENO1 + PP: 0.51823, 0.43721, 0.47533

Over ENO1: 0.79782, 0.71852, 0.76205

Empty Vector + PP: 0.39826, 0.47672, 0.43985

Empty Vector: 0.88152, 0.79863, 0.83565

Table 6 data

AKT

Over ENO1 + PS: 0.987413205, 0.918293205, 0.849173205

Over ENO1 + PP: 0.888042685, 0.829611685, 0.771180685

Empty Vector + PP: 0.691275845, 0.620075845, 0.548875845

C-MYC

Over ENO1 + PS: 0.622259536, 0.555159536, 0.488059536

Over ENO1 + PP: 0.471387946, 0.413087946, 0.354787946

Empty Vector + PP: 0.32229694, 0.26469694, 0.20709694

Table 7 data

ERBB2

Over ENO1 + PS: 0.879771008, 0.810571008, 0.741371008

Over ENO1 + PP: 0.703556759, 0.632056759, 0.560556759

Empty Vector + PP: 0.43630023, 0.38530023, 0.33430023

PI3K

Over ENO1 + PS: 0.781839631, 0.712529631, 0.643219631

Over ENO1 + PP: 0.614610857, 0.556490857, 0.498370857

Empty Vector + PP: 0.466944415, 0.414034415, 0.361124415

CyclinE1

Over ENO1 + PS: 0.787680784, 0.737470784, 0.687260784

Over ENO1 + PP: 0.658924335, 0.613724335, 0.568524335

Empty Vector + PP: 0.39095006, 0.35084006, 0.31073006

Table 8 data

ENO1

Over ENO1 + PS: 0.756563905, 0.706463905, 0.656363905

Over ENO1 + PP: 0.648700796, 0.608500796, 0.568300796

Empty Vector + PP: 0.447219382, 0.397109382, 0.346999382

MMP2

Over ENO1 + PS: 0.871624498, 0.808914498, 0.746204498

Over ENO1 + PP: 0.716721538, 0.666311538, 0.615901538

Empty Vector + PP: 0.538215806, 0.470903806, 0.403591806
