# Supplementary material for: Pine pollen reverses the function of hepatocellular carcinoma by inhibiting α-Enolase mediated PI3K/AKT signaling pathway
Source: PLoS One. 2024 Nov 22;19(11):e0312434. doi: 10.1371/journal.pone.0312434 (PMC11584142; doi:10.1371/journal.pone.0312434)
Supplement: Attachment — Submitted filename: Review-Comments-PlosOne.docx [file pone.0312434.s006.docx]

Luo et al. presented a study applying various computational and experimental analysis to investigate the biological functions of Pine pollen in hepatocellular carcinoma. They found that Pine pollen inhibits HCC via ENO1, MBP-1 and PI3K/Akt pathway. The analysis and results appear intriguing and promising; however, there are several major issues in the current manuscript that undermine the study's clarity and conclusion.

1. The manuscript's English writing is quite poor, which significantly affects its readability. For example, on page 2 line 39, it is confusing to read “We analyzed the bioinformatics of ENO1”, which should be revised to “We applied various bioinformatic analysis of open-access data to study the expression of ENO1”. It is recommended that the authors seek assistance from a professional editing service or a native English speaker to enhance the quality of the writing.
2. The structure of the paper is currently disorganized, such that there are nine figures and seven tables totally, making it difficult for readers to follow the authors’ arguments and findings. The authors should reorganize the figures/tables to provide a coherent flow of information, such as putting some figures into supplementary file. Also, the index in Figure 3 is incorrect, please revise that.
3. The statistical analysis presented in the manuscript is not adequately justified and appears unreasonable in parts. For example, on page 18 line 184, the authors claimed that they applied one-way ANOVA test; however, at least in Figure 2 the test should be t-test or Wilcoxon test depends on the distribution of data. Besides, is the p-value in this study adjusted? The authors should revisit their analytical methods to ensure they are appropriate for their data.
4. The interpretation of the figures in the manuscript needs to be more accurate and detailed. Each figure and panel should be clearly explained. For example, how does each part in Figure 4 support the conclusion “Establishment of stable cell line overexpressing ENO1”?

Overall, the current manuscript cannot be considered for publication. The authors should improve the quality and clarity substantially so that the manuscript can meet the publication standards.
