# Supplementary material for: Pine pollen reverses the function of hepatocellular carcinoma by inhibiting α-Enolase mediated PI3K/AKT signaling pathway
Source: PLoS One. 2024 Nov 22;19(11):e0312434. doi: 10.1371/journal.pone.0312434 (PMC11584142; doi:10.1371/journal.pone.0312434)
Supplement: Attachment — Submitted filename: Response to Reviewers.docx [file pone.0312434.s007.docx]

1. The English has been polished by authoritative institutions.
2. The table of cell scratch experiment has been included as a supplementary document. The index of Figure 3 has been modified.
3. The inter group analysis with time overlap adopts multiple analysis of variance. The numerical change was misread at the time, and the P-value has not been changed. I consulted with statistical experts, and they believe that this is correct.
4. The chart has been reorganized: Figure 4 (A) White light and fluorescence photos of overexpressed ENO1 cells transfected for 72 hours, with fluorescence cells accounting for approximately 80% of the white light cell count; Fluorescence represented successfully transfected cells. (B) White light and fluorescence photos of empty vector cells transfected for 72 hours, with fluorescent cells accounting for approximately 80% of the white light cell count; Fluorescence represented successfully transfected cells. (C) White light and fluorescence photos of non transfected cells cultured for 72 hours, but the cells did not show fluorescence; (D) Photos of overexpressed ENO1 cells screened with 20 μg/μl purinomycin for 3 days showed good cell growth; photos of empty transfected cells screened with 20 μg/μl purinomycin for 3 days showed good cell growth; and photos of non transfected cells screened with 20 μg/μl purinomycin for 3 days showed that all cells were killed by puromycin; Screening with puromycin involves killing untransfected cells that are not resistant to puromycin, while screening stable transfected cells that are resistant to puromycin. (E) RT-qPCR dissolution curve of overexpression ENO1 cells showed that the amplification product was ENO1; The histogram of relative expression of ENO1 RT-qPCR in overexpression ENO1 cells and empty vector cells showed the expression level of ENO1 in overexpressing ENO1 transfected cells was 25.09 times higher than that in empty transfected cells. These results indicated that ENO1 could be overexpressed in overexpressing ENO1 transfected cells.

Figure 5 (A) shows that the empty transfected cells were cultured in wells without PP solution for 12 hours as a negative control; Overexpression stable transformed cells were cultured in 5 μ L PP solution wells for 12 hours; Overexpression stable transformed cells were cultured in 75 μ L PP solution wells for 12 hours; (B) The empty transfected cells were cultured in wells without PP solution for 24 hours as a negative control; Overexpression stable transformed cells were cultured in 5 μ L PP solution wells for 24 hours; Overexpression stable transformed cells were cultured in 75 μ L PP solution wells for 24 hours. This indicates that compared to the negative control, as the concentration of PP solution increases, the degree of inhibition of overexpression stable transformed cells becomes more severe. (C) The relationship between the logarithm of drug concentration and the corresponding cell inhibition rate of PP solution after 12 hours of action was plotted. The calculated IC50 was 5.78 μ g/μ l, which was used as the PP solution concentration and corresponding action time for subsequent experiments.
