# Supplementary material for: Pine pollen reverses the function of hepatocellular carcinoma by inhibiting α-Enolase mediated PI3K/AKT signaling pathway
Source: PLoS One. 2024 Nov 22;19(11):e0312434. doi: 10.1371/journal.pone.0312434 (PMC11584142; doi:10.1371/journal.pone.0312434)
Supplement: Attachment — Submitted filename: Response to Reviewers.docx [file pone.0312434.s008.docx]

1. The manuscript's English writing is quite poor, which significantly affects its readability. For example, on page 2 line 39, it is confusing to read “We analyzed the bioinformatics of ENO1”, which should be revised to “We applied various bioinformatic analysis of open-access data to study the expression of ENO1”. It is recommended that the authors seek assistance from a professional editing service or a native English speaker to enhance the quality of the writing.

Answer: The English has been polished by authoritative institutions.

2. The structure of the paper is currently disorganized, such that there are nine figures and seven tables totally, making it difficult for readers to follow the authors’ arguments and findings. The authors should reorganize the figures/tables to provide a coherent flow of information, such as putting some figures into supplementary file. Also, the index in Figure 3 is incorrect, please revise that.

Answer: The table of cell scratch experiment has been included as a supplementary document. The index of Figure 3 has been modified.

3. The statistical analysis presented in the manuscript is not adequately justified and appears unreasonable in parts. For example, on page 18 line 184, the authors claimed that they applied one-way ANOVA test; however, at least in Figure 2 the test should be t-test or Wilcoxon test depends on the distribution of data. Besides, is the p-value in this study adjusted? The authors should revisit their analytical methods to ensure they are appropriate for their data.

Answer: Multivariate analysis of variance is used for intergroup analysis with temporal overlap in statistics. The numerical change was misread at the time, and the P-value has not been changed. I consulted with statistical experts, and they believe that this is correct.

4. The interpretation of the figures in the manuscript needs to be more accurate and detailed. Each figure and panel should be clearly explained. For example, how does each part in Figure 4 support the conclusion “Establishment of stable cell line overexpressing ENO1”?

Answer: The chart has been reorganized: Figure 4 and Figure 4 (A) show the white light and fluorescence images of cells overexpressing ENO1 after transfection for 72 hours, with fluorescent cells accounting for approximately 80% of the white light cells; Fluorescence represents successfully transfected cells. (B) White light and fluorescence photos of empty vector cells transfected for 72 hours, with fluorescent cells accounting for approximately 80% of the white light cells; Fluorescence represents successfully transfected cells. (C) White light and fluorescence photos of untransfected cells cultured for 72 hours, but the cells did not show fluorescence; (D) After screening cells overexpressing ENO1 with 20 μ g/μ l puromycin for 3 days, the photos showed good cell growth; After screening empty vector transfected cells with 20 μ g/μ l puromycin for 3 days, the photos showed good cell growth; After screening untransfected cells with 20 μ g/μ l puromycin for 3 days, the photos showed that all cells were killed by puromycin; Screening with puromycin involves killing untransfected cells that are intolerant to puromycin, while selecting stable transfected cells that are tolerant to puromycin. (E) The RT qPCR dissolution curve of overexpressing ENO1 cells showed that the amplified product was ENO1; The histogram of relative expression levels of ENO1 RT qPCR in overexpressing ENO1 cells and empty vector cells showed that the expression level of ENO1 in overexpressing ENO1 transfected cells was 25.09 times higher than that in empty vector transfected cells. These results indicate that ENO1 can be overexpressed in cells transfected with ENO1.

Figure 5 (A) shows that the empty transfected cells were cultured in wells without PP water extract for 12 hours as a negative control; Overexpression stable transformed cells were cultured in 5 μ L PP water extract wells for 12 hours; Overexpression stable transformed cells were cultured in 75 μ L PP water extract wells for 12 hours; (B) The empty transfected cells were cultured in wells without PP water extract for 24 hours as a negative control; Overexpression stable transformed cells were cultured in 5 μ L PP water extract wells for 24 hours; Overexpression stable transformed cells were cultured in 75 μ L PP water extract wells for 24 hours. This indicates that compared to the negative control, as the concentration of PP water extract increases, the degree of inhibition of overexpression stable transformed cells becomes more severe. (C) The relationship between the logarithm of drug concentration and the corresponding cell inhibition rate of PP solution after 12 hours of action was plotted. The calculated IC50 was 5.78 μ g/μ l, which was used as the PP solution concentration and corresponding action time for subsequent experiments.

Reviewer's comment response:

1. When preparing the “Response To Reviewer Comments”, the authors should copy and paste the original comments from last round and then provide the point-by-point response to each comment. This is to maintain an easy and straightforward tracking of all comments and responses. Also, as the revision guidelines indicated, the authors should upload a marked-up copy of manuscript that highlights changes made to the original version (upload this as a separate file labeled 'Revised Manuscript with Track Changes')

Answer: We have responded to the comments one by one and provided a manuscript with revision marks, named "Revised Manuscript".

2. “The English has been polished by authoritative institutions.” The Englishwriting has indeed improved in current submission; However, there are still some sentences appear incorrect, such as in Abstract, Conclusion, the authors wrote “PP inhibits HCC by regulating the expression of ENO1 and MBP-1 and inhibiting the 53 PI3K/AKT pathway by inhibiting C-MYC and erb-B2 receptor tyrosine kinase 2.” It is confusing to read all three “inhibiting”, please revise that to make it clearer and more concise.

Answer: The second "inhibiting" in "PP inhibitors HCC by regulating the expression of ENO1 and MBP-1 and inhibiting the PI3K/AKT pathway by inhibiting C-MYC and erb-B2 receptor tyrosine kinase 2." has been changed to "suppressing".

3. The format of reference numbers is incorrect. If the numbers are listed as superscript, then there should not be brackets around them. Please revise that and follow the official format by the journal.

Answer: The superscript of the reference number has been changed to normal.
